# Memory Effects in Nanolaminates of Hafnium and Iron Oxide Films Structured by Atomic Layer Deposition

**DOI:** 10.3390/nano12152593

**Published:** 2022-07-28

**Authors:** Kristjan Kalam, Markus Otsus, Jekaterina Kozlova, Aivar Tarre, Aarne Kasikov, Raul Rammula, Joosep Link, Raivo Stern, Guillermo Vinuesa, José Miguel Lendínez, Salvador Dueñas, Helena Castán, Aile Tamm, Kaupo Kukli

**Affiliations:** 1 Institute of Physics, University of Tartu, W. Ostwaldi 1, 50411 Tartu, Estonia; markus.otsus@ut.ee (M.O.); jekaterina.kozlova@ut.ee (J.K.); aivar.tarre@ut.ee (A.T.); aarne.kasikov@ut.ee (A.K.); raul.rammula@ut.ee (R.R.); aile.tamm@ut.ee (A.T.); kaupo.kukli@ut.ee (K.K.); 2 Laboratory of Chemical Physics, National Institute of Chemical Physics and Biophysics, Akadeemia tee 23, 12618 Tallinn, Estonia; joosep.link@kbfi.ee (J.L.); raivo.stern@kbfi.ee (R.S.); 3 Department of Electronics, University of Valladolid, Paseo Belén 15, 47011 Valladolid, Spain; guillermo.vinuesa@alumnos.uva.es (G.V.); josemilen99@gmail.com (J.M.L.); sduenas@ele.uva.es (S.D.); helcas@tel.uva.es (H.C.)

**Keywords:** multilayers, atomic layer deposition, hafnium oxide, iron oxide, ferromagnetism, resistive switching, nanolaminates

## Abstract

HfO_2_ and Fe_2_O_3_ thin films and laminated stacks were grown by atomic layer deposition at 350 °C from hafnium tetrachloride, ferrocene, and ozone. Nonlinear, saturating, and hysteretic magnetization was recorded in the films. Magnetization was expectedly dominated by increasing the content of Fe_2_O_3_. However, coercive force could also be enhanced by the choice of appropriate ratios of HfO_2_ and Fe_2_O_3_ in nanolaminated structures. Saturation magnetization was observed in the measurement temperature range of 5–350 K, decreasing towards higher temperatures and increasing with the films’ thicknesses and crystal growth. Coercive force tended to increase with a decrease in the thickness of crystallized layers. The films containing insulating HfO_2_ layers grown alternately with magnetic Fe_2_O_3_ exhibited abilities to both switch resistively and magnetize at room temperature. Resistive switching was unipolar in all the oxides mounted between Ti and TiN electrodes.

## 1. Introduction

Prospective applications to magnetoresistive [1] and resistive [2] memory effects, which were notified few decades ago, have extended the search for potentially multiferroic materials, whereby the list of such materials has continuously been updated. Studies on layered compound materials, concurrently exhibiting resistive switching (RS) and ferromagnetic (FM) characteristics, have been conducted. Both RS and FM properties could have simultaneously been registered in devices built on thin films of few different compounds, e.g., ZnO:Co [3], ZnO:Co/SiO_2_:Co [4], copper oxides [5], or HfO_2_ [6]. Aside from nonlinear, saturative, and hysteretic magnetization behavior, bipolar resistive switching behavior was recorded in devices built on these compounds, expressed by two distinct conduction current (resistivity) states stabilized during consecutive programming pulses upon changes in voltage polarity.

The films referred to above were formed by physical vapor deposition (PVD) techniques. The exploitation of PVD allows the formation of solid films with maximum chemical purity, as the purity of a deposited material is determined by that of the precursor sublimed. At the same time, PVD techniques may face challenges before the uniform deposition of thin films over substrates of arbitrary area and shape. Chemical vapor deposition routes to the multifunctional ferroic films are thus additionally sought in order to open complementary perspectives to tailor materials possessing useful, and differently manifested, physical properties. Amongst several materials that exhibit magnetoresistive performance, HfO_2_ can be regarded as a material feasibly grown over large area substrates if appropriate routes, such as atomic layer deposition (ALD), were used. HfO_2_ might further stand out as a compound exhibiting resistive switching behavior together with magnetic susceptibility, especially when supported by an additive or dopant-enhancing internal magnetization, particularly Fe_2_O_3_.

HfO_2_, together with Ta_2_O_5_, has been considered one of the most intensely investigated thin film materials for application in nonvolatile, resistively switching (memristive) memories, which are based on the migration of either cations or oxygen vacancies into the lattice of a switching medium [7]. The resistive switching (RS) effect is a reversible and non-volatile change in the resistance of a material. By applying a certain electric field, a conductive filament (CF) is formed through the oxide, connecting the metals that surround it. Once the filament is formed for the first time (electroforming process), different electric field values will allow for repetitively disrupting (RESET process) and forming (SET process) the CF reversibly [2,8,9]. If the SET and RESET processes occur at different voltage polarities, the effect is known as bipolar resistive switching (BRS), which is usually due to oxygen anion migration and electron hopping through oxygen vacancies in the oxide media (valence change mechanism or VCM) whereby the metal electrodes are inert metals [2,8,10,11,12,13]. BRS can also be produced by the formation of a metallic filament (electrochemical metallization mechanism or ECM, also known as conductive bridge RAM or CBRAM). In the latter case, an electrochemically active metal electrode is needed as a source of the metal cations that will diffuse through the oxide, creating the CF [2,8,10,11,12,13]. On the other hand, unipolar resistive switching (URS) is attributed to thermochemical processes dominating over electrochemical ones (thermochemical memory or TCM) [14,15]. Thus, temperature gradients produced by Joule heating lead to redox reactions and local changes in the material (oxide) stoichiometry, which results in a change of the conductivity [10,11,12,13]. The switching mechanism in oxide film media, including HfO_2_, has largely been described as that based on filamentary conduction [16]. Nonetheless, switching in HfO_2_ has also been found to be dependent on electrode metals, whereby both bipolar and unipolar switching could be initiated [17]. Resistively switching HfO_2_ films can be synthesized using different techniques, including metalorganic chemical vapor deposition [17] and atomic layer deposition (ALD) [18]. 

The application of external magnetic fields can influence the resistive switching performance of ALD-processed HfO_2_ based cells [18]. Further, internal magnetization in HfO_2_ films alone has been possible due to the presence of significant amounts of oxygen vacancies [19,20], which may also be related to the formation of metastable HfO_2_ polymorphs. In general, the achievement and appearance of ferromagnetic-like behavior has been regarded as an inherent, although sometimes unexpected, property of nanocrystalline materials [21]. Nonlinear hysteretic magnetization is in such cases caused by defective crystallite boundaries involving vacancies and unsaturated coordination of metal atoms, i.e., the factors generally causing leakage and increasing conductivity. Thereby, the magnetization should take place in a medium that, at the same time, should also switch resistively. The prerequisite for the resistive switching process is insulation in the virgin, although defective, state of the medium. Thus, the demands for materials demonstrating both magnetization and resistive switching may appear controversial. Such materials should in principle simultaneously perform as wide-band-gap dielectrics and electrically rather conductive magnetic materials, which may be regarded as contradicting properties. Therefore, a tradeoff between insulating dielectric properties and the ability to hysteretically magnetize upon choosing the constituent materials is necessary. Engineering combinations of reliably resistively switching material layers, such as HfO_2_, with complementary compounds reliably magnetizing and also growing, such as Fe_2_O_3_ [22,23,24], is justified and is to be purposefully examined in terms of both memory effects. Quite naturally, before the examination of coupling between hysteretic magnetization and electrical switching, it would be reasonable to examine the appearance of both effects separately, in order to attempt optimization of the deposition process and the forming structures. This is essentially the aim of the present study.

Studies on composites or solid solutions based on iron and hafnium oxides are scarce. Crystallization has been investigated in the HfO_2_-Fe_2_O_3_ system [25] at temperatures more than two times higher than those applied in the present study. The enhancement of magnetization has been observed in Fe-doped HfO_2_ [26] due to the phase segregation and formation of Fe_2_O_3_. Composite materials, especially in the form of functional thin films consisting of iron and hafnium oxides, are thus not quite explored yet. 

Regarding layered materials containing either HfO_2_ or Fe_2_O_3_, we have earlier observed both saturative hysteretic magnetization and bipolar resistive switching behavior in HfO_2_-Al_2_O_3_ [27], HfO_2_-ZrO_2_ [28,29], and SiO_2_-Fe_2_O_3_ [30] multilayers grown by atomic layer deposition. In the present study, nonlinear saturative and hysteretic magnetization in HfO_2_-based thin solid films grown by ALD, enhanced by the contribution from Fe_2_O_3_ to HfO_2_-based multistoried films, was examined. Here, HfO_2_ and Fe_2_O_3_ layers were grown sequentially into stacks to tailor magnetic and insulating materials. The goal was to ensure nonlinear hysteretic magnetization as well as resistive switching in the same materials, yet without a detailed investigation of the coupling effects that remain beyond the scope of the present study. The objective was to examine whether it is possible to observe reliable switching behavior and hysteretic magnetization at room temperature in HfO_2_-Fe_2_O_3_ nanolaminates grown using the same deposition cycle sequences.

## 2. Materials and Methods

The films studied in this work were grown in a low-pressure flow-type ALD reactor [31]. Hafnium tetrachloride (HfCl_4_, 99.9%, Sigma Aldrich, Burlington, MA, USA), used as the hafnium precursor, and ferrocene (Fe(C_5_H_5_)_2_, 99.5%, Alfa Aesar, Word Hill, MA, USA), used as an iron precursor, were evaporated at 160 and 83 °C, respectively, from a half-open glass boat inside the reactor. Nitrogen (N_2_, 99.999%, AS Linde Gas, Tallinn, Estonia) was applied as the carrier and purging gas. Ozone produced from O_2_ (99.999% purity, AS Linde Gas) was used as an oxidizer. The ALD reactions were carried out at 350 °C. Cycle times for Fe_2_O_3_ deposition were 5-5-5-5 s for the sequence: metal precursor pulse—N_2_ purge—O_3_ pulse—N_2_ purge, respectively. Cycle times for HfO_2_ were 5-2-5-5 s for an analogous sequence. Single HfO_2_ and Fe_2_O_3_ films were grown to thicknesses ranging from 20 to 60 nm in order to acquire the reference data from composition analysis as well as resistive switching or magnetizing media. Further, a double-layered Fe_2_O_3_-HfO_2_ stack as well as nanolaminates of HfO_2_ and Fe_2_O_3_ were deposited, aiming at the formation of a series of stacks consisting of both iron-rich and hafnium-rich solid media, as presented in Table 1. 

The films were grown on undoped Si(100) and, for the electrical evaluation, also on highly doped conductive Si substrates covered by a 10 nm TiN film. The conductive Si wafers were boron-doped to concentrations of 5 × 10^18^ − 1 × 10^19^ cm^–3^ and coated with crystalline TiN layer by pulsed chemical vapor deposition using a batch TiCl_4_/NH_3_ process [32,33] at temperatures of 450–500 °C in an ASM A412 Large Batch 300 mm reactor at Fraunhofer IPMS-CNT. The films, which were deposited on TiN substrates for electrical measurements, were also supplied with Ti/Au electron-beam evaporated electrodes on top of the films, with the Ti layer directly contacting the switching oxide medium and Au deposited in order to provide non-oxidizing electrical contact to the measurement circuit.

The crystal structure of the films was evaluated by grazing incidence X-ray diffraction (GIXRD) method using a SmartLab (Rigaku, Tokyo, Japan) X-ray diffractometer and the CuKα radiation with a wavelength of 0.15406 nm. The same apparatus was exploited to determine the thickness, density, and roughness of the films by X-ray reflectometry (XRR). Energy dispersive X-ray spectrometry (EDS) measurements were carried out at an accelerating voltage of 15 kV with a current of 0.69 nA using an INCA Energy 350 EDS spectrometer (Oxford Instruments, Abingdon, Oxfordshire, UK) connected to a Helios Nanolab 600 (FEI) scanning electron microscope. Scanning transmission electron microscopy (STEM) and elemental mapping of the films in cross-sectional orientation were performed in a Cs-corrected Titan Themis 200 microscope (FEI, Hillsboro, OR, USA). EDS maps were acquired using Esprit software version 1.9 (Bruker, Billerica, MA, USA). Thin cross-sectional samples for STEM observations were prepared using the in situ lift-out technique using a Helios Nanolab 600 scanning electron microscope/focused ion beam system (FEI, Hillsboro, OR, USA), equipped with a super-X EDX system (FEI/Bruker). In order to protect the surface from ion milling during the preparation of STEM samples, the area of interest was covered with a platinum protection layer. An X-ray fluorescence (XRF) analyzer ZSX400 (Rigaku) was complementarily used for the elemental composition analysis. Considering the feasibility of the measurements, the composition analysis was conducted on Fe_2_O_3_ and HfO_2_ reference films grown to somewhat higher thicknesses using 500 ALD cycles, whereas thinner films grown using 200 cycles to thicknesses similar to those of nanolaminates were further subjected to electrical and magnetic measurements.

Electrical measurements were carried out in a probe station using a Keithley 4200-SCS semiconductor analyzer (Keysight Technologies, Cleveland, OH, USA). In the DC measurements, the bias voltage was applied to the top electrode, and the bottom electrode remained grounded. To initiate RS, every sample required an electroforming procedure that was carried out as a voltage sweep with positive bias and a current compliance of 10 µA to avoid irreversibly breaking the device. Capacitance measurements were performed by applying an AC signal of 30 mV along with a DC bias of 0.1 V in the frequency range 1–1000 kHz. Magnetic measurements were performed using the Vibrating Sample Magnetometer (VSM) option of the Physical Property Measurement System 14T (Quantum Design, San Diego, CA, USA) by scanning the magnetic field from −1.5 to 1.5 T parallel to the film surface in the temperature range of 5–350 K.

## 3. Results and Discussion

### 3.1. Growth and Structure

**Figure 1:**
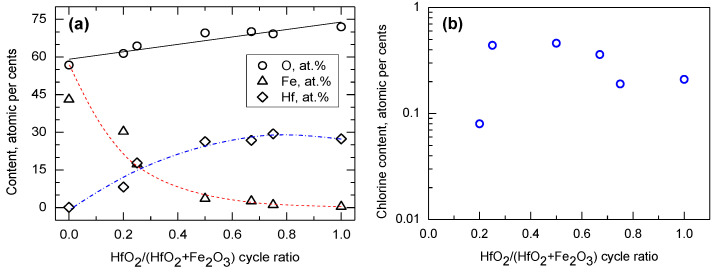
Contents of elements (**a**) and residual impurities (**b**) measured by XRF, constituting the HfO_2_-Fe_2_O_3_ films, expressed in atomic% against relative amount of HfO_2_ deposition cycles. The elements are indicated in the legends. Polynomial lines are guides for the eye.

The metal oxide films constituting the nanolaminates were grown in processes under the same reactor conditions and exploiting the same precursor chemistry as those earlier suited to the growth of HfO_2_ [34] and Fe_2_O_3_ [35] films. In the present study, the thickness of multilayered HfO_2_-Fe_2_O_3_ films could be appreciably well controlled by adjusting the amounts of the growth cycles for constituent oxide layers (Table 1). In addition, the presence of iron and hafnium in the films was proven by both XRF and EDS analysis, whereby the relative contents of both metals were correlated to the relative amounts of their growth cycles applied (Table 1, Figure 1a). Further, chlorine and carbon were detected as impurities present in the films (Figure 1b). Both impurities can be regarded as natural residues arising from the ligands to the metal precursors. Within the accuracy limits of the analysis method, the content of Cl and C was not systematically dependent on the relative deposition cycle ratio.

**Figure 2:**
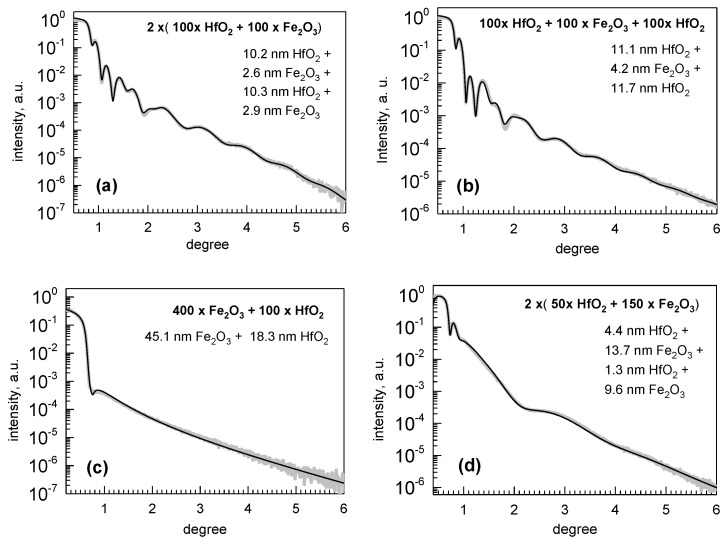
X-ray reflectivity results for selected stacks of Fe_2_O_3_ and HfO_2_ layers grown on Si, denoted by the labels revealing the amounts of ALD cycles used for the deposition of constituent layers. The thicknesses of constituent layers as the results of the curve fittings are also given by labels. The curves with fitting results are presented for the four-layer laminate grown using equal amounts of cycles for both constituent oxides (**a**), the three-layer laminate grown using equal amounts of cycles for both constituent oxides (**b**), the double-layer consisting of relatively thicker Fe_2_O_3_ and thinner HfO_2_ films (**c**), and the four-layer laminate containing Fe_2_O_3_ layers relatively thicker compared to the HfO_2_ component (**d**).

The HfO_2_-Fe_2_O_3_ multilayers were truly formed as nanolaminates, as proven by XRR results depicted in Figure 2. The X-ray reflection intensity curves allowed one to fit the measured data, in a good approximation, with the predicted thicknesses of the constituent layers of both metal oxides, roughly correlated to the amounts of deposition cycles applied in the case of both HfO_2_ and Fe_2_O_3_. It is well known that in the case of ALD, the growth rates of materials on substrates of foreign composition may markedly differ at different growth stages, generally being essentially lower at the early stages of growth, i.e., at low thicknesses. The influence of the nucleation rate and the length of the so-called incubation period depends on temperature, substrate, and growing material, and should be explored separately, if required. In the present study, the growth rate of HfO_2_ on Fe_2_O_3_ noticeably exceeded the growth rate of Fe_2_O_3_ on HfO_2_. In the case of the growth of HfO_2_ on crystallized Fe_2_O_3_ (Figure 2c), the growth rate of HfO_2_ could reach as high as 0.18 nm/cycle, possibly supported by a larger specific surface area of underlying polycrystalline iron oxide layer.

**Figure 3:**
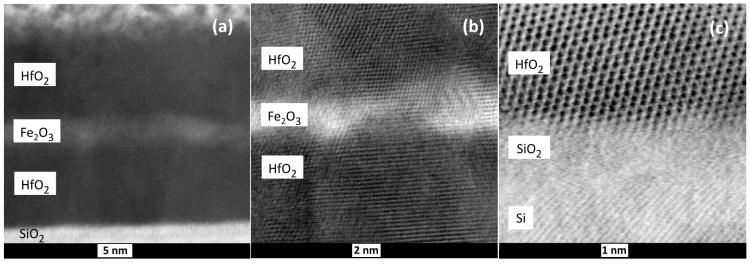
Bright field STEM images of HfO_2_-Fe_2_O_3_-HfO_2_ nanolaminate grown using 100 ALD cycles for each constituent layer, taken under different magnifications (**a**,**b**), and an image of the interface between silicon substrate and the first HfO_2_ layer in the same laminate (**c**).

**Figure 4:**
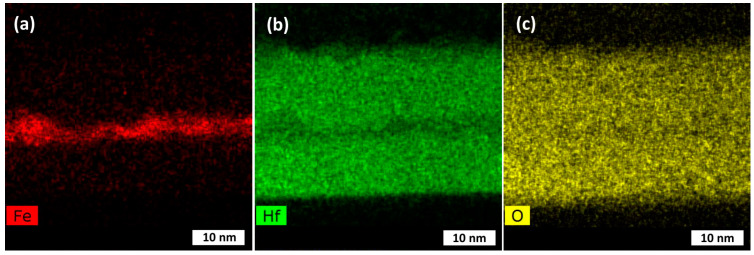
Elemental mapping for iron (**a**), hafnium (**b**), and oxygen (**c**) in the HfO_2_-Fe_2_O_3_-HfO_2_ nanolaminate grown using 100 ALD cycles for each constituent layer.

STEM studies revealed sequential deposition of the hafnium and iron oxide layers, distinct in terms of structure (Figure 3) and elemental distribution (Figure 4), thus supporting the XRR results. One can see that the layers were crystallized throughout the film thickness (Figure 3a,b) without structurally sharp interfaces between the HfO_2_ and Fe_2_O_3_ constituent layers, still enabling the distinction between metal oxides due to the differences in atomic numbers. At the same time, the interface between HfO_2_ and the amorphous SiO_2_ top layer on the Si substrate was sharply formed and distinct (Figure 3c). It is, however, to be noted that the growth rates and resulting thicknesses of component layers in nanolaminate structures are not to be compared to those of reference films. It is well-known that the films grown by ALD require an incubation time before achieving stable growth and structural formation. The length of the incubation period may vary considerably, depending on the material to be grown as well as the substrate material and structure. Nucleation at early stages, i.e., growth of HfO_2_ on Fe_2_O_3_ and vice versa would require a separate study. Compositionally, the constituent layers became clearly distinguishable (Figure 4), allowing one to rely on the formation of nanomaterial composed of physically and chemically different oxides, further enabling the appearance of both magnetic and insulating materials’ properties.

**Figure 5:**
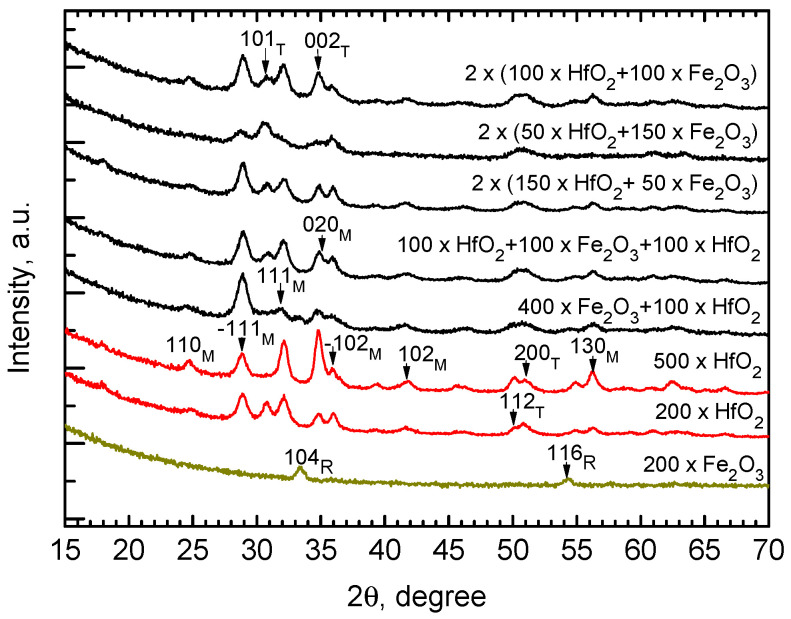
Grazing incidence X-ray diffraction patterns of Fe_2_O_3-_, HfO_2-_, and HfO_2_-Fe_2_O_3_-laminated films. The growth cycle sequences are denoted by labels. The reflections supplied with Miller indexes are assigned as those belonging to either monoclinic (M, ICDD PDF-2 card no 43-1017) or tetragonal (T, card 01-078-5756) HfO_2_, whereby reflections from rhombohedral hematite Fe_2_O_3_ are denoted by R (card 01-1053).

The Fe_2_O_3_ films grown alone without alternate layering with HfO_2_ were moderately crystallized in their as-deposited states (Figure 5, the bottom pattern). Two weak but still distinct reflection peaks at 33.6 and 56.5° could be attributed to the 104 and 116 reflections of rhombohedral Fe_2_O_3_, that is, the hematite phase. At the same time, the HfO_2_ films grown alone without alternate layering with Fe_2_O_3_ were relatively more crystallized in their as-deposited states (Figure 5, the 2nd pattern from bottom). The HfO_2_ film grown using 200 deposition cycles could be described as a multiphase solid medium consisting of a stable monoclinic phase of HfO_2_ and a metastable, quite likely tetragonal, polymorph of HfO_2_. Whereas most of the reflection peaks remained very weak, the most distinct reflections unambiguously belonging to the −111 and 111 of monoclinic HfO_2_ peaked at 28.3 and 31.5°, respectively. Between the latter reflections, a peak assigned as 101 of tetragonal HfO_2_ was clearly detected at 30.3°. These three neighboring reflections can be regarded as proof of multiphase composition. Further and notably, after 500 ALD cycles, the metastable phases, if initially formed and present in HfO_2_ films, were already almost insignificant in the diffraction patterns. The diffractogram from the HfO_2_ film grown using 500 cycles comprised reflections attributed exclusively to monoclinic HfO_2_ (Figure 5, the 3rd pattern from bottom).

Somewhat surprisingly, in the diffractograms taken from most of the films consisting of stacked HfO_2_ and Fe_2_O_3_ layers, no reflections attributable to any of the known iron oxide phases could be recognized. At the same time, crystallization in the films was obvious and due to the crystal growth in the HfO_2_ corresponding to the stacks or multilayers. With regard to the reflections characteristic of HfO_2_, the significance of the ones attributable to −111 and 111 of monoclinic HfO_2_, peaking at 28.3 and 31.5°, respectively, tended to increase with the relative amounts of HfO_2_ deposition cycles (Figure 5). The relative significance of the reflection assigned as 101 of tetragonal HfO_2_, at 30.3°, quite expectedly increased with the decrease in the relative amount of the HfO_2_ deposition cycles (Figure 5). Notably, reflections characteristic of Fe_2_O_3_ did not appear in the stacked HfO_2_ and Fe_2_O_3_ layers, with an exception of the sample grown using the cycle sequence of 400 × Fe_2_O_3_ + 100 × HfO_2_, where a weak 104 peak of rhombohedral hematite phase could be recognized at 33.5°. 

### 3.2. Magnetization Behavior

**Figure 6:**
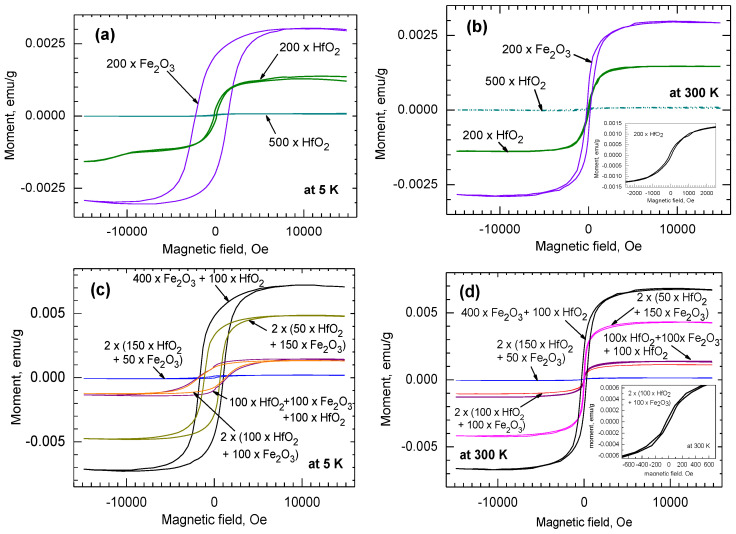
Magnetization-field curves from reference HfO_2_ and Fe_2_O_3_ films measured at 5 K (**a**) and at 300 K (**b**), as compared to the curves from HfO-Fe_2_O_3_-laminated structures measured at 5 K (**c**) and 300 K (**d**). The films were grown on SiO_2_/Si substrates using cycle sequences represented by labels.

The reference Fe_2_O_3_ film grown on diamagnetic Si substrates expectedly demonstrated ferromagnetic-like magnetization behavior (Figure 6a) with the coercive force measured as strong as 2272 Oe at 5 K. The thinnest 24 nm-thick HfO_2_ films grown in the present study using 200 ALD cycles were magnetized nonlinearly and saturatively in external fields (Figure 6a). Both the saturation magnetization and the coercive field remained low, although clearly measurable, in the 24 nm-thick HfO_2_ film compared to those of the Fe_2_O_3_ films. At the same time, the saturation magnetization and coercivity in the 54 nm-thick HfO_2_, grown using 500 cycles, became suppressed almost entirely. This is plausibly due to the obvious difference between phase compositions of 24 and 54 nm-thick HfO_2_ films (Figure 5). The thinner HfO_2_ film contained, besides stoichiometric monoclinic HfO_2_, probably also oxygen-deficient metastable either tetragonal or cubic HfO_2_, which would give rise to the magnetization. Upon an increase in the film thickness and crystal growth, the formation of dominant monoclinic dioxide caused a suppression of the magnetization in the solid material. At room temperature, the saturation magnetization values were not significantly decreased, differently from coercitivities. Upon increasing the measurement temperatures from 5 to 300 K, the coercivities were decreased nearly 10, 4, and 2 times in the Fe_2_O_3_ film grown using 200 and 500 cycles, and in HfO_2_ film grown using 200 cycles, respectively (Figure 6b)

With regard to the HfO_2_ films, undoped and crystallized hafnium dioxide is not supposed to magnetize in its bulk and stoichiometric form, and contamination by handling with stainless-steel tweezers may sometimes have led to measurable magnetic signals [36]. However, magnetization earlier unexpectedly detected in HfO_2_ films [37] can intentionally be induced, as supported by the presence of defects, in the first place oxygen vacancies [19,20]. Oxygen vacancies are inevitable constituents in the metal oxide lattices, also considered as a cause of the filamentary switching mechanism [38,39,40] in the device cells. 

In the samples in which Fe_2_O_3_ and HfO_2_ films were grown into double, triple, or four-layered stacks, the saturation magnetization values obtained at both 5 and 300 K tended, somewhat expectedly, to be the highest in the samples where the amount of sequential Fe_2_O_3_ growth cycles exceeded those applied for the HfO_2_ by 3–5 times (Figure 6c). However, in the case of laminated films, one should take into account that the microstructure of the individual Fe_2_O_3_ layers, most strongly affecting the magnetic properties, also varies due to the thickness variation (Figure 2). For instance, in the double-layered (400 × Fe_2_O_3_ + 100 × HfO_2_) sample, the Fe_2_O_3_ was moderately crystallized, whereas in all other laminated samples, Fe_2_O_3_ was X-ray-amorphous (Figure 5). Notably, the thickness of Fe_2_O_3_ in the double layer was nearly 10 times higher compared to that in the three- and four-layer samples when 100 cycles of Fe_2_O_3_ was applied (see Figure 2a,b). Apparently, in the latter samples, smaller Fe_2_O_3_ nanocrystals could form and possibly agglomerate. The coercivity value measured at 5 K for the film consisting of two double layers of HfO_2_ and Fe_2_O_3_ grown using 50 and 150 cycles, respectively, exceeded 1100 Oe. Coercivity in the double layer consisting of a Fe_2_O_3_ film grown at first using 400 cycles, followed by HfO_2_ grown using 100 cycles, reached nearly 1500 Oe. At room temperature, these values were decreased down to 65 and 271 Oe, respectively.

**Figure 7:**
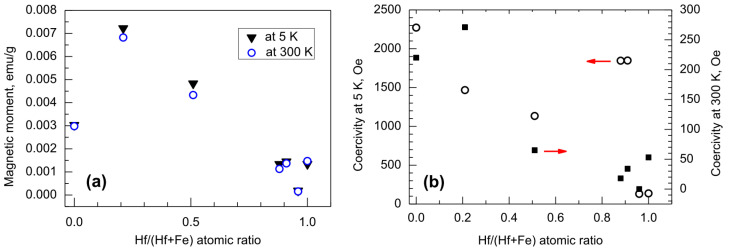
Magnetic moment (**a**) and coercivity (**b**) against relative content of hafnium, expressed by Hf/(Hf + Fe) cation ratio, in HfO_2_-Fe_2_O_3_ nanolaminates. For the cycle ratios and sequences applied for the growth of the samples with the corresponding atomic ratios, see Table 1.

Despite the ability of nanocrystalline HfO_2_ to moderately magnetize, also revealing hysteretic performance, the magnetization in terms of both saturation and coercive force was quite naturally dominated by Fe_2_O_3_ constituting the nanolaminates. Higher amounts of iron in the iron-hafnium oxide multilayers certainly caused increments in both saturation magnetization (Figure 7a) and coercivity (Figure 7b). Interestingly, relatively strong coercivities among nanolaminate samples, reaching nearly 1850 Oe at 5 K and remaining below 50 Oe at 300 K, were obtained in the triple HfO_2_-Fe_2_O_3_-HfO_2_ layer and in four-layer film consisting of two HfO_2_-Fe_2_O_3_ double layers (Figure 6c). These samples were characterized by Hf/(Hf + Fe) ratios of 0.91 and 0.88, respectively (Figure 7b, Table 1). In the latter two samples, all the constituent metal oxide layers were grown using 100 ALD cycles. At the same time, the saturation magnetization in the same samples was decreased about five times below the values characterizing the films containing Fe_2_O_3_ layers grown using 150 and 400 cycles, described above. It is thus possible that despite the relatively low Fe_2_O_3_ amount in such laminates and accompanying weak saturation magnetization, the growth of constituent oxides in such nanolaminates after application of sufficient amounts of deposition cycles has enabled the ordering and growth of nanocrystals enhancing structure or shape anisotropy and a simultaneous increment in coercivity. Further and more detailed studies including parametrization and scaling up the process would be required in order to clarify the interdependencies between deposition cycle numbers, crystallographic orientation, and magnetic performance. 

**Figure 8:**
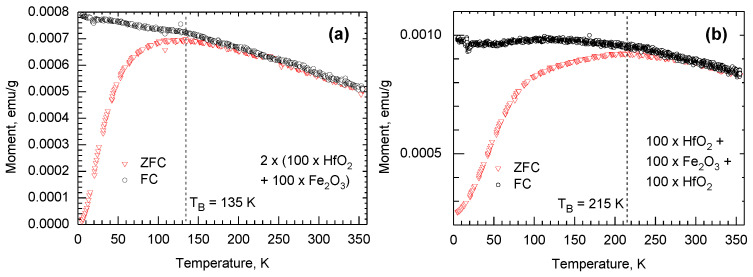
Magnetization-temperature curves measured in zero-field-cooling (ZFC) and field-cooling (FC) mode from representative structures consisting of two HfO_2_-Fe_2_O_3_ double layers (**a**) and one HfO_2_-Fe_2_O_3_-HfO_2_ triple layer (**b**). The films were grown on diamagnetic SiO_2_/Si substrates using cycle sequences represented by labels.

The samples based on triple HfO_2_-Fe_2_O_3_-HfO_2_-layer and four-layer film consisting of two HfO_2_-Fe_2_O_3_ double layers are those worth further attention, because the same samples further exhibited defined resistive switching behavior, as will be described below. Superparamagnetic behavior was expected at first, and the same samples were additionally subjected to the temperature-dependent magnetization measurements. As was already implied by the magnetization-field curves recorded at 5 and 300 K (Figure 6c,d), the triple-layered film grown using the cycle sequence of 100 × HfO_2_ + 100 × Fe_2_O_3_ + 100 × HfO_2_ exhibited slightly higher saturation magnetization values compared to the four-layered film grown using the cycle sequence of 2 × (100 × HfO_2_ + 100 × Fe_2_O_3_). This can plausibly be explained by the distribution of Fe_2_O_3_ differing in the latter two samples. In the four-layered sample, the thickness of the two Fe_2_O_3_ layers summarized was similar to that in the three-layer sample containing a single Fe_2_O_3_ layer in between two HfO_2_ layers (Figure 2). Therefore, in the three-layer sample, the short-range order in Fe_2_O_3_, although not detectable by GIXRD, could possibly be better defined compared to that in the four-layer film, providing higher saturation magnetization. Further, field-cooling (FC) measurements were carried out in the temperature range of 355–5 K (Figure 8), recording the magnetization values at each temperature after the application of the external field of 1000 Oe. For both samples, the zero-field-cooling (ZFC) measurements were also carried out in order to estimate the blocking temperatures related to thermal energy, below which the magnetization in a material consisting of single-domain nanocrystallites would lose its preferred direction in a near-zero external field and relax. In the four-layer structure (Figure 8a), the blocking temperature, T_B_, remained lower compared to that estimated for the three-layer structure (135 vs. 215 K, Figure 8b). This may be indicative of the effect of the lower amount of easily magnetized, and for this reason, also more easily reoriented, amount of iron-rich nanoparticles present in the three-layer film.

### 3.3. Electrical Behavior

**Figure 9:**
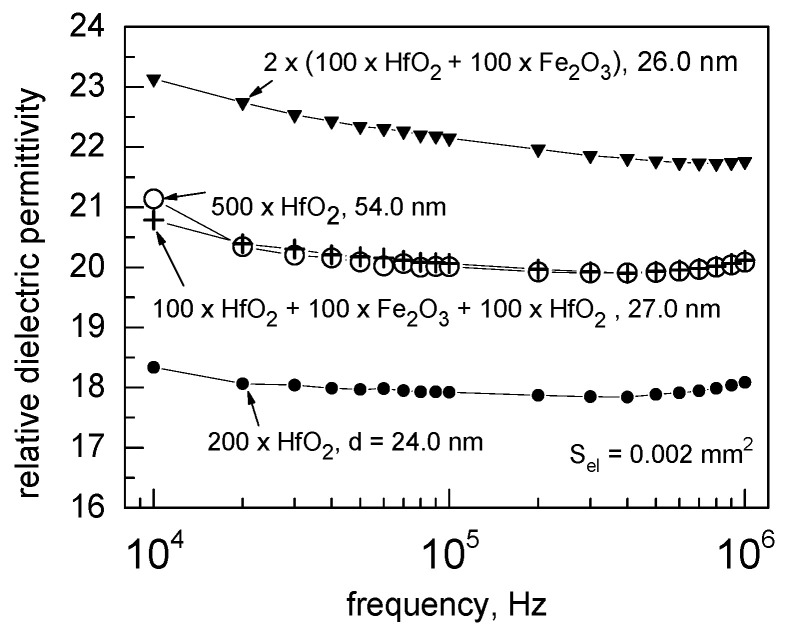
Permittivity versus measurement frequency dispersion test results for reference HfO_2_ films as well as nanolaminate stacks grown using amounts of ALD cycles and to the thicknesses indicated by the labels. The capacitor electrode areas were 0.002 mm^2^.

The reference HfO_2_ films behaved electrically as dielectric materials with defined insulating properties. Figure 9 represents the results of capacitance dispersion measurements, expressed as permittivity-frequency curves, after calculating the permittivity considering the simple parallel-plate capacitor configuration, containing dielectric film with thickness determined by XRR measurements. One can see that the permittivity-frequency dependencies represent appreciably flat plateaus, being indicative of the minor role for parasitic space charge and interfacial polarization in the frequency range of 10 kHz–1 MHz. The average permittivity value for the 24 nm-thick HfO_2_ film grown using 200 deposition cycles remained at 18, whereas the permittivity of the 54 nm-thick film grown using 500 cycles exceeded 20. This difference was plausibly due to the more developed crystallinity in the thicker film. Since the HfO_2_ films in the present study were characterized with multiphase composition (Figure 5), i.e., contained both metastable and stable polymorphs of HfO_2_, the permittivity value remained even, quite expectedly, between those earlier calculated as those characteristic of corresponding phases [41]. One could also note that the permittivity values measured in the present study corresponded appreciably well to those measured earlier in a study reporting insulating properties of HfO_2_-based nanolaminates and single HfO_2_ films grown by ALD using HfCl_4_ and H_2_O as precursors [42].

The HfO_2_-Fe_2_O_3_-HfO_2_ triple layer and the periodically deposited sample consisting of two HfO_2_-Fe_2_O_3_ double layers grown using 100 deposition cycles for each component oxide behaved electrically reliable enough to enable stable capacitance measurements. The capacitance, and consequently the estimated permittivity values, appeared higher than those in the reference HfO_2_ film grown to the comparable thickness (Figure 9), possibly due to the influence of interfacial polarization enabled by the relatively leaky intermediate Fe_2_O_3_ layers. Furthermore, even the iron-rich sample consisting of periodical nanolaminate deposited using the cycle sequence of 2 × (50 × HfO_2_ + 150 × Fe_2_O_3_) film grown to the total thickness of 29 nm occurred electrically stable enough, exhibiting permittivity values up to 50–60 (not shown) in the frequency range examined. Iron oxide films, deposited without complementary HfO_2_ layers, could not be capacitively evaluated due to their high conductivity. One could clearly see that alternate layering of HfO_2_ and Fe_2_O_3_ usefully assisted the trade-off between conductivity and capacitance increments, being indicative of the possibility to usefully tune the electrical resistivity in magnetic materials. The reliability of capacitance measurements, revealing the permittivity of the artificially structured HfO_2_-Fe_2_O_3_ laminates, evidently governed by the properties of HfO_2_, allows one to further expect stability of the resistive switching phenomena. 

**Figure 10:**
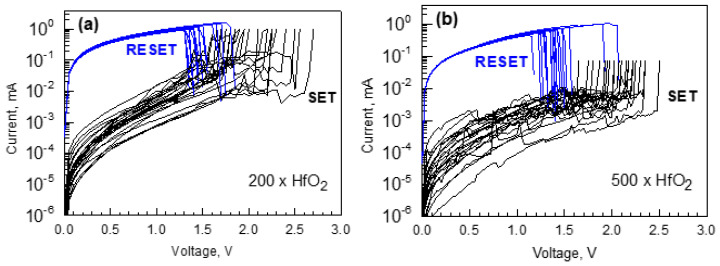
Current-voltage characteristics demonstrating unipolar switching in TiN/HfO_2_/Ti devices containing HfO_2_ films grown to thicknesses of 24 (**a**) and 54 nm (**b**) using 200 and 500 ALD cycles, respectively.

The HfO_2_ reference films grown to the thicknesses of 24 and 54 nm after application of 200 and 500 deposition cycles, respectively, exhibited well-defined unipolar resistive switching behavior (Figure 10). The main RS parameters, such as low to high resistive state ratios and switching voltages, did not differ markedly for the films grown to different thicknesses, being indicative of the insensitivity of the polymorphic phase composition differing in these HfO_2_ films (Figure 5). Switching parameters did not depend on the device area in those samples in which both 0.002 mm^2^ and 0.052 mm^2^ electrode surface areas delivered RS behavior. In the case of the HfO_2_ films grown to the thickness of 54 nm, reliable switching behavior could be observed only upon measurements conducted on the electrodes of the smallest size, 0.002 mm^2^ (Figure 10b), whereas in the case of the HfO_2_ films grown to the thickness of 24 nm, electrodes with an area of 0.052 mm^2^ also allowed one to acquire reliable switching characteristics (Figure 10a). Plausibly, growing film thickness enables the growth of larger crystallites accompanied by higher surface roughness, structural inhomogeneities, and voids, necessitating measurements on smaller sized electrodes in order to acquire reliable results. One has to note that an apparent dependence of switching reliability on electrode area is not necessarily to be related to the domination of the interfacial conduction mechanism over the filamentary one. Indeed, the unipolar switching produced by the thermochemical mechanism (TCM) is known to be filamentary [14,43], and the switching parameters, i.e., resistance in both the LRS and HRS as well as current values, are expected to be independent from the electrode area [14,44,45,46]. Under this thermochemical mechanism, thermochemical redox processes dominate the electrochemical ones. Local redox reactions occur due to a local temperature increase that energetically favors lower oxidation states. Thus, oxygen drifts out of this high-temperature region, producing a variation of the local conductivity due to stoichiometry variations, which are usually attributed to the formation of a lower oxidation state sub-oxide, or the metal itself if such sub-oxide does not exist [15,47]. The filaments can then be induced under the chosen voltage polarity and disrupted afterwards by the currents causing Joule heating, healing the defective channel under an applied voltage of the same polarity, but inducing much higher currents in the low resistivity state. As it is common in cells presenting unipolar resistive switching, the low to high resistivity state ratios could exceed three orders of magnitude, with a high cycle-to-cycle variability of the switching voltages and the resistance value of the HRS.

**Figure 11:**
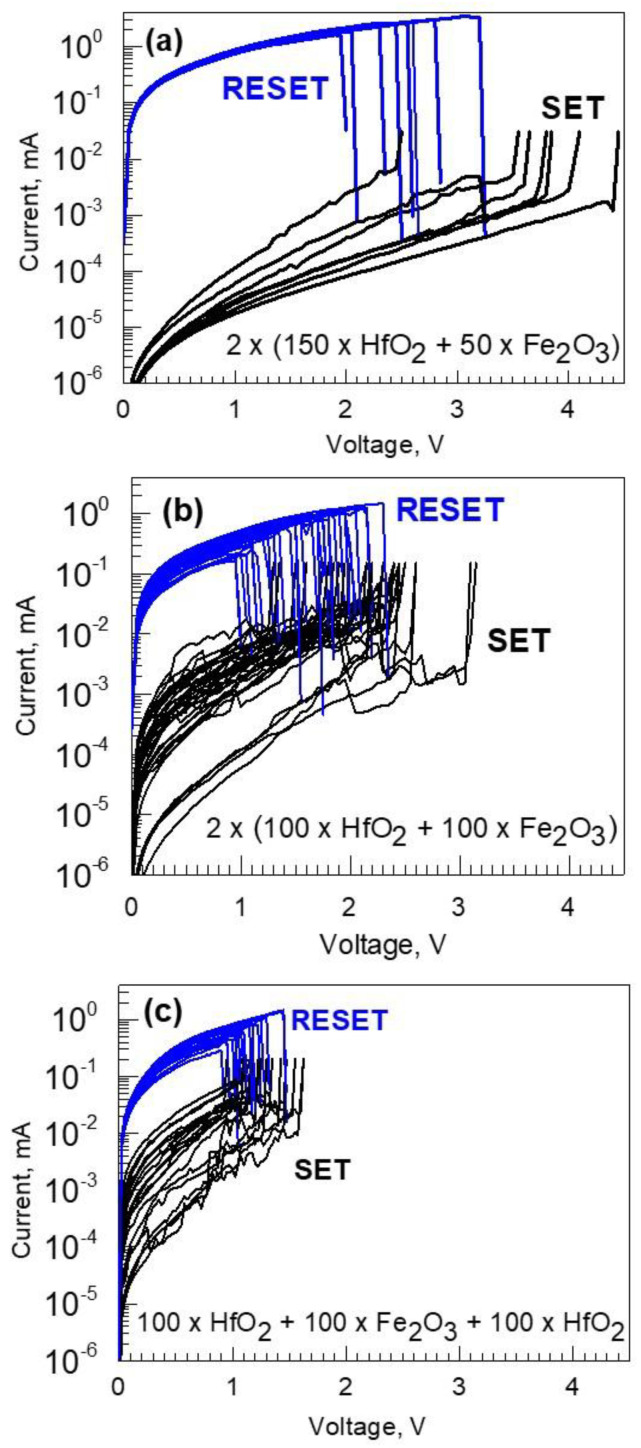
Current-voltage characteristics demonstrating unipolar switching in TiN/HfO_2_-Fe_2_O_3_/Ti devices containing (**a**) periodically laminated media grown using relatively large amounts of HfO_2_ deposition cycles, (**b**) periodically laminated media grown using the same cycle numbers for HfO_2_ and Fe_2_O_3_, and (**c**) three-layer stack with Fe_2_O_3_ layer grown in between HfO_2_ films. The deposition cycle sequences are indicated by labels.

Figure 11 demonstrates defined switching behavior recorded in periodical HfO_2_-Fe_2_O_3_ laminates (Figure 11a,b) as well as in the HfO_2_-Fe_2_O_3_-HfO_2_ triple-layer stack (Figure 11c). It is worth noting that in the periodically stacked HfO_2_-Fe_2_O_3_ laminate grown using the cycle sequence of 2 × (150 × HfO_2_ + 50 × Fe_2_O_3_), the low to high resistivity state ratios could reach even five orders of magnitude (Figure 11a). However, the repeatability of switching cycles in such films remained rather low, not allowing one to reliably record more than 8–10 switching cycles. To additionally recall, this sample was relatively weakly magnetized (Figure 6c,d and Figure 7), demonstrating almost insignificant saturation magnetization and rather weak coercitivity (below 200 Oe) already at 5 K compared to the rest of the samples grown using higher relative amounts of Fe_2_O_3_ deposition cycles. Thus, the deposition program applied to this particular sample could result in a material representing trade-off between magnetic and resistively switching performance, implying a possibility for optimization. 

**Figure 12:**
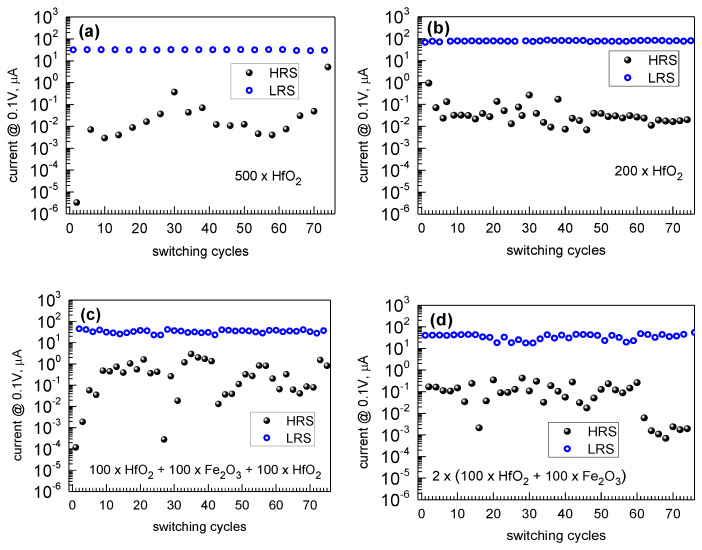
Current values measured after each RESET (HRS points) and SET (LRS points) processes at 0.1 V in TiN/HfO_2_-Fe_2_O_3_/Ti devices containing (**a**) 500 and (**b**) 200 ALD cycles of HfO_2_, (**c**) three-layer stack with Fe_2_O_3_ layer grown in between HfO_2_ films, and (**d**) periodically laminated media grown using the same cycle numbers for HfO_2_ and Fe_2_O_3_. The deposition cycle sequences are indicated by labels.

The repeatability of current-voltage behavior upon resistive switching cycles after endurance tests was obvious in the reference HfO_2_ films (Figure 12a,b). However, noticeable scattering in the current values in high-resistance states upon sequential switching cycles was recorded. One could suppose that the stability of switching the current level in the HRS after the RESET events is markedly dependent of the structural quality of the material restored after breaking filaments or quenching the conduction channels. At the same time, the resistivity levels in LRS after SET events might more significantly be determined by the current compliance limits. In this regard, the stability of HRS could serve as a structure-related descriptor of the crystalline switching medium and be worth depicting as an implication of the device functionality. 

The decrement in the low to high resistivity ratio was detected simultaneously with increased variability in low resistivity states. The LRS:HRS ratios during endurance tests remained slightly higher and also somewhat more stable in the reference HfO_2_ films (Figure 12a,b) compared to the results recorded in a device built on triple-layered HfO_2_-Fe_2_O_3_-HfO_2_ film (Figure 12c) as well as on four-layered HfO_2_-Fe_2_O_3_-HfO_2_-Fe_2_O_3_ (Figure 12d), all grown to comparable thicknesses. The increased variability in the LRS is most plausibly due to the iron oxide constituents in the films, increasing the material conductivity already in its virgin state before the SET event. This property may ease the formation of conductive channel but worsen the control over breaking it. The main outcome of the RS measurements was, thus, the recognition of a certain balance between growth cycles of constituent dielectric and magnetic materials while engineering the deposition process, before the achievement of both resistive switching and magnetic polarization at room temperature.

It should be emphasized that the unipolar resistive switching behavior of these samples has been of high interest when studying the physics behind RS. Although previous studies on HfO_2_-based RRAM devices have also shown unipolar resistive switching, it was in some way always due to the metals chosen for the bottom and top electrodes [48]. It is known that RRAM cells that use the same metal for both electrodes will most likely show URS [13], as observed in the case of Pt/HfO_2_/Pt [49,50] and TiN/HfO_2_/TiN [51] MIM stacks. At the same time, Pt/HfO_2_/TiN cells can demonstrate both URS and BRS [52,53]. Pt/HfO_2_/Ti stacks have also shown bipolar switching [49], indicating better properties of Ti as an oxygen reservoir when compared to TiN, enabling and supporting the VCM. Thus, the use of a thin Ti cap in TiN/HfO_2_/Ti/TiN devices allows one to observe BRS [54], with unipolar switching appearing only under extreme programming conditions [55]. When looking at RRAM cells similar to the ones presented in this work, i.e., HfO_2_-based RRAM with TiN and Ti as bottom and top electrodes (BE an TE), respectively, the literature strongly suggests that bipolar switching is expected [48,49,50], contrary to the results presented here. This could be attributed to the use of Au as the top electrode over to Ti. Bertaud et al. [56] showed that the use of a metal with low a enthalpy of formation of oxides, such as gold, for the TE, should lead to unipolar switching, and Walzcyk et al. [57] reported URS in Au/HfO_2_/TiN MIM stacks. Here, it is worth noting that a previous work on resistively switching media based on Fe_2_O_3_ has also reported URS [58], although their stack made use of somewhat symmetric Pt metal electrodes (Pt/Fe_2_O_3_/Pt/Ti). Nevertheless, a study conducted as early as 1969 [59] reports unipolar switching in thin iron oxide, associating increased conductivity (forming or SET processes) either with the formation of sub-oxides mentioned above [47] or with the expected decrease in transition metals’ resistance with increasing temperature, known as metal-insulator transitions [60], which has been specifically proven for both thick [61] and thin Fe_2_O_3_ films [62].

## 4. Summary and Conclusions

Insulating HfO_2_ and magnetic Fe_2_O_3_ were successfully tailored as nanolaminates of alternately layered films of hafnium and iron oxides. The relative contents of hafnium and iron were modified by changing the relative amounts of the deposition cycles for both constituent oxides. The films contained some amounts of chlorine and carbon as residual impurities. The structure of the films could be regarded as nanocrystalline, whereby a multiphase nature of nanolaminates, based on the simultaneous appearance of both stable and metastable polymorphs of HfO_2_, was established. 

Stable dielectric polarization and resistive switching properties expectedly characteristic of HfO_2_ were achieved and recorded in the laminated stacks containing measurable amounts of relatively highly conducting Fe_2_O_3_. At the same time, the presence of Fe_2_O_3_ as well as formation of nanocrystalline phases of HfO_2_ enabled the appearance and recording of nonlinear, saturative, and hysteretic magnetization in the laminates, somewhat correlated with the relative content of Fe_2_O_3_. The coercivity appeared the strongest in the films consisting of two sequentially grown HfO_2_-Fe_2_O_3_ double layers as well as a triple HfO_2_-Fe_2_O_3_-HfO_2_ layer, measured at 5 K. The coercivity diminished drastically at room temperature, remaining, however, measurable below 100 Oe. Notably, the same structures, clearly also exhibited an ability to switch resistively, whereas samples containing relatively larger amounts of iron could not switch resistively due to increasing conductivity. 

All samples deposited using 100 or less Fe_2_O_3_ cycles between HfO_2_ layers demonstrated unipolar resistive switching. Higher amounts of Fe_2_O_3_ ALD cycles resulted in a decrease of the low to high resistance state ratio. Fe_2_O_3_ layers constituting the laminates probably did not allow for the formation of conductive filaments as efficiently as HfO_2_, hindering the variability of the low resistance state. The appearance of the unipolar resistive switching of these samples may be regarded as notable in light of common knowledge suggesting that Ti and TiN used for the top and bottom electrodes, mounting HfO_2_, should lead to bipolar resistive switching. This could be due to the dominance of a thermochemical process over an electrochemical one. Further studies could be devoted to the investigation of the effect of electrodes with variable enthalpy as well as coupling between conductivity and electromagnetic polarization.

## Figures and Tables

**Table 1:** List of HfO_2_, Fe_2_O_3_, and HfO_2_-Fe_2_O_3_ samples revealing the deposition cycle sequences, relative HfO_2_/(HfO_2_ + Fe_2_O_3_) deposition cycle ratio, total film thickness in accord with XRR, and relative Hf/(Hf + Fe) cation ratio measured by XRF and EDS. The samples are presented in the order of decreasing relative cycle ratio for HfO_2_ and, concurrently, descending cation ratio for Hf, measured by XRF. For the contents of residual chlorine, also measured by XRF, see Figure 1.

Cycle Sequence	Cycle Ratio	t_total_, nm	Hf/(Hf + Fe) by XRF	Hf/(Hf + Fe) by EDS
500 × HfO_2_	1	54	1	1
2 × (150 × HfO_2_ +50 × Fe_2_O_3_)	0.75	23	0.96 (0.03)	0.98
100 × HfO_2_ + 100 × Fe_2_O_3_ + 100 × HfO_2_	0.67	27	0.91 (0.04)	0.95
2 × (100 × HfO_2_ +100 × Fe_2_O_3_)	0.5	26	0.88 (0.02)	0.91
2 × (50 × HfO_2_ +150 × Fe_2_O_3_)	0.25	29	0.51 (0.01)	0.49
400 × Fe_2_O_3_ +100 × HfO_2_	0.2	63	0.21 (0.01)	0.24
500 × Fe_2_O_3_	0	37	0	0

## Data Availability

Not applicable.
